# Maternal, delivery and neonatal outcomes in women with cervical cancer. A study of a population database

**DOI:** 10.18632/oncoscience.613

**Published:** 2025-01-20

**Authors:** Aaron Samuels, Ahmad Badeghiesh, Haitham Baghlaf, Michael H. Dahan

**Affiliations:** ^1^McGill Faculty of Medicine and Health Sciences, McGill University, Montreal, Quebec, Canada; ^2^Department of Obstetrics and Gynecology, King Abdulaziz University, Rabigh, Saudi Arabia; ^3^Division of Maternal-Fetal Medicine, Department of Obstetrics and Gynecology, University of Tabuk, Tabuk, Saudi Arabia; ^4^Department of Obstetrics and Gynecology, Division of Reproductive Endocrinology, McGill University Health Center, Royal Victoria Hospital (Glen Site), Montreal, Quebec H4A 3J1, Canada

**Keywords:** cervical cancer, pregnancy, fetal outcomes, maternal outcomes, delivery outcomes

## Abstract

Importance: Cervical cancer is the fourth most common cancer among women globally and a significant cause of cancer-related deaths. Understanding the impact of cervical cancer diagnosed during pregnancy on maternal, delivery, and neonatal outcomes is crucial for improving clinical management and outcomes for affected women and their children.

Objective: To determine the effects of cervical cancer diagnosed during pregnancy on maternal, delivery, and neonatal outcomes using a population based, American database.

Design: This study is a retrospective analysis of the Healthcare Cost and Utilization Project Nationwide Inpatient Sample (HCUP-NIS) database. The study period spans between 2004–2014, and the analysis was conducted in 2023.

Setting: The study used the HCUP-NIS database, which includes data from hospital stays across the United States, covering 48 states and the District of Columbia.

Participants: The study included all women who delivered a child or had a maternal death from 2004–2014, with pregnancies at 24 weeks or above. The population was comprised of 9,096,788 pregnant women, including 222 diagnosed with cervical cancer prior to delivery.

Exposures: The exposure was a diagnosis of cervical cancer during pregnancy, identified using International Classification of Diseases 9th Revision codes 180.0, 180.1, 180.8, and 180.9.

Main Outcomes and Measures: Primary outcomes included maternal, delivery, and neonatal complications including preterm delivery, cesarean section, hysterectomy, blood transfusion, deep venous thrombosis, pulmonary embolism, congenital anomalies, intrauterine fetal demise, and small-for-gestational-age neonates. Logistic regression analyses were conducted to evaluate the association between cervical cancer diagnosis and these outcomes, adjusting for potential confounding factors.

Results: Women with cervical cancer were older (25.2% ≥35 years vs. 14.7%, *p* = 0.001, respectively); more likely to have Medicare insurance (1.4% vs. 0.6%, *p* = 0.005, respectively); use illicit drugs (4.1% vs. 1.4%, *p* = 0.001, respectively); smoke tobacco during pregnancy (14.9% vs. 4.9%, *p* = 0.001, respectively); and have chronic hypertension (3.6% vs. 1.8%, *p* = 0.046, respectively). When controlling for confounding effects women with cervical cancer had higher rates of preterm delivery (aOR = 4.73, 95% CI (3.53–6.36), *p* = 0.001); cesarean section (aOR = 5.40, 95% CI (4.00–7.30), *p* = 0.001); hysterectomy (aOR = 390.23, 95% CI (286.43–531.65), *p* = 0.001); blood transfusions (aOR = 19.23, 95% CI (13.57–27.25), *p* = 0.001); deep venous thrombosis (aOR = 9.42, 95% CI (1.32–67.20), *p* = 0.025); and pulmonary embolism (aOR = 20.22, 95% CI (2.83–144.48), *p* = 0.003). Neonatal outcomes, including congenital anomalies, intrauterine fetal demise, and small-for-gestational-age neonates, were comparable between groups.

Conclusions and Relevance: Cervical cancer during pregnancy is associated with significant maternal and delivery risks, however, neonatal outcomes are largely unaffected. These findings highlight the need for a multidisciplinary approach in managing pregnant cervical cancer patients, involving oncological, obstetrical, and neonatal care specialists.

## INTRODUCTION

Cervical cancer ranks as the fourth most common cancer among women globally and remains a leading cause of cancer-related deaths. In 2022, approximately 660,000 new cervical cancer cases were reported, resulting in 350,000 deaths [1]. The primary risk factor for cervical cancer is Human Papillomavirus (HPV) infection [2]. Additionally, other risk factors include smoking [2], having multiple sexual partners [2], and initiating sexual intercourse at a young age [2], most of which contribute to an increased susceptibility to HPV infection. As such, regular pap smears are indicated in women for the early detection of cervical abnormalities. The staging of cervical cancer is determined using the FIGO (International Federation of Gynecology and Obstetrics) system, which evaluates tumor size, lymph node involvement, and distant metastasis. This staging guides treatment decisions, which can range from surgical interventions like hysterectomy for early-stage disease to radiotherapy and chemotherapy for advanced stages.

The incidence of cervical cancer among pregnant women ranges from 1.4 to 4.6 per 100,000 and is rising due to recent trends of delayed marriage and later childbearing [3]. However, there is a scarcity of data documenting the maternal, delivery and neonatal consequences of cervical cancer during pregnancy. Cervical cancer diagnosed during pregnancy can be linked with adverse effects, which can either arise from the cancer’s inherent characteristics or from the interventions used to treat it [4]. A study on preserving pregnancy in cervical cancer patients found that among a group of 40 women diagnosed with cervical cancer while pregnant, there were no significant survival differences between the pregnancy continuation and termination groups [3]. Moreover, surgical outcomes were found to be similar between the two groups [3]. In that same study they determined that neoadjuvant chemotherapy did not substantially harm fetal health [3]. However, it’s important to note that the small sample size of this study (*n* = 40) may limit its reliability. Another meta-analysis that combined data from 15 studies examined conception rates in women with a history of cervical cancer, concluding that conception rates are not negatively impacted in women with an intact uterus who were previously treated for cervical cancer [5].

Cervical cancer diagnosed in pregnancy may result in the initiation of chemo or radiation therapy causing both maternal, placenta and fetal effects. It may also require pre-term delivery to prevent further advancement of the carcinoma. Cesarean section may be indicated in women with significant cervical cancers to prevent intra partum and post-partum hemorrhage.

Given the potential effects of cervical cancer during gestation and the paucity of relevant studies, it is critical to gather additional information on this topic to better understand pregnancy risks. Our study aims to address the knowledge gap regarding the outcomes of cervical cancer on both maternal and fetal health. Utilizing a comprehensive contemporary nationwide database, we seek to determine the effects of cervical cancer during pregnancy on maternal, delivery, and neonatal outcomes.

## RESULTS

A total of 9,096,788 pregnant women met the inclusion criteria. Of them, 222 patients were diagnosed with cervical cancer prior to delivery.

Table 1 lists the demographic and baseline characteristics of pregnant women with and without a diagnosis of cervical cancer prior to delivery. Compared to those without, women with a cervical cancer diagnosis prior to delivery were characterized by higher maternal age (25.2% ≥35 years vs. 14.7%, *p* = 0.001, respectively); increased likelihood to have Medicare insurance (1.4% vs. 0.6%, *p* = 0.005, respectively); higher rate of illicit drug use (4.1% vs. 1.4%, *p* = 0.001, respectively) increased likelihood of smoking tobacco during pregnancy (14.9% vs. 4.9%, *p* = 0.001, respectively); and increased likelihood of having chronic hypertension (3.6% vs. 1.8%, *p* = 0.046, respectively). Other maternal characteristics, such as race, income quartiles, obesity, previous cesarean section, pregestational diabetes mellitus, multiple gestation, thyroid disease, HIV infection, and *in-vitro* fertilization treatments to conceive the pregnancy, were comparable between the two groups.

**Table 1:** Comparing maternal characteristics between cervical cancer and non-cervical cancer groups

Maternal characteristics			
Characteristics	Cervical cancer *N* = 222 (%)	No cervical cancer *N* = 9096566 (%)	*P*-value
Age (years)			0.001
<25	43 19,4%	3455812 38,0%	
25–34	123 55,4%	4299780 47,3%	
≥35	56 25,2%	1340964 14,7%	
Race			0.085
White	119 53,6%	4482540 49,3%	
Black	35 15,8%	1649349 18,1%	
Hispanic	51 23,0%	2029277 22,3%	
Asian and Pacific	<11	441810 4,9%	
Native American	<11	64144 0,7%	
Other	<11	365654 4,0%	
Income quartiles			0.172
Less than 39,000	65 29,3%	2218075 24,4%	
$39,000–47,999	70 31,5%	3080060 33,9%	
$48,000–62,999	64 28,8%	2416072 26,6%	
$63,000 or more	23 10,4%	1382307 15,2%	
Meical Insurance Plan type			0.005
Medicare	<11	56600 0,6%	
Medicaid	113 50,9%	3882663 42,7%	
Private including HMO	87 39,2%	4606886 50,6%	
Self-pay	13 5,9%	288423 3,2%	
No charge	0 0,0%	17062 0,2%	
Other	<11	244932 2,7%	
Obesity	<11	324167 3,6%	0.693
Previous CS	39 17,6%	1452451 16,0%	0.515
Tobacco Smoking during pregnancy	33 14,9%	443557 4,9%	0.001
Chronic HTN	<11	165222 1,8%	0.046
Pregestational DM	<11	86611 1,0%	0.192
Illicit Drug use	<11	125610 1,4%	0.001
Multiple gestation	<11	137301 1,5%	0.457
Thyroid disease	<11	223275 2,5%	0.288
HIV	0 0,0%	2079 0,0%	0.822
IVF	0 0,0%	10532 0,1%	0.879

Table 2 displays the association between a cervical cancer diagnosis prior to delivery and pregnancy outcomes, delivery outcomes and other uncategorizable outcomes after adjusting for potential confounders, including age, medical insurance plan type, illicit drug use, chronic hypertension, and tobacco smoking during pregnancy. Pregnancy outcomes such as pregnancy-induced hypertension, gestational hypertension, preeclampsia, eclampsia, hypertension and superimposed preeclampsia/eclampsia, gestational diabetes mellitus, and placenta previa were comparable between women who were diagnosed with cervical cancer prior to delivery and those who were not. In terms of delivery outcomes, women with a cervical cancer diagnosis prior to delivery were found to have higher rates of preterm delivery (OR = 4.73, 95% CI (3.53–6.36), *p* = 0.001); cesarean section (OR = 5.40, 95% CI (4.00–7.30), *p* = 0.001); hysterectomy (OR = 390.23, 95% CI (286.43–531.65), *p* = 0.001); and blood transfusions (OR = 19.23, 95% CI (13.57–27.25), *p* = 0.001). Lower rates of operative vaginal deliveries (OR = 0.27, 95% CI (0.09–0.84), *p* = 0.024); and spontaneous vaginal deliveries (OR = 0.21, 95% CI (0.16–0.29), *p* = 0.001) were noted in the CC group. Other delivery outcomes examined such as preterm premature abruption of membranes, postpartum hemorrhage, wound complications, maternal death, placental abruption, chorioamnionitis, delivery, were comparable between the two groups. Regarding other outcomes, women with a cervical cancer diagnosis prior to delivery were found to have higher rates of deep venous thrombosis (OR = 9.42, 95% CI (1.32–67.20), *p* = 0.025) and pulmonary embolisms (OR = 20.22, 95% CI (2.83–144.48), *p* = 0.003), but were comparable to the control group concerning maternal infection, venous thromboembolism, and disseminated intravascular coagulation.

**Table 2:** Comparing pregnancy and delivery outcomes between cervical cancer and non-cervical cancer groups

Pregnancy and delivery outcomes					
Outcomes	Cervical cancer (N) (%)	No cervical cancer (N) (%)	Crude OR (95% CI)	Adjusted OR (95% CI)	Adjusted *p*-value
**Pregnancy outcomes^a^**							
Pregnancy induced hypertension	13 5.9%	673736 7.4%	0.78 (0.44–1.36)	0.77 (0.44–1.35)	0.363
Gestational hypertension	<11	301603 3.3%	0.54 (0.20–1.44)	0.57 (0.21–1.52)	0.259
Preeclampsia	<11	327383 3.6%	0.87 (0.41–1.85)	0.91 (0.43–1.94)	0.814
Eclampsia	0 0%	6944 0.1%	NA	NA	0.996
Preeclampsia and Eclampsia superimposed HTN	<11	47363 0.5%	1.74 (0.43–6.99)	0.99 (0.22–4.56)	0.989
GDM	22 9.9%	523170 5.8%	1.80 (1.16–2.80)	1.44 (0.92–2.42)	0.111
Placenta previa	<11	49979 0.5%	2.48 (0.794–7.75)	1.95 (0.62–6.10)	0.252
**Delivery outcomes^b^**												
PPROM	<11	103616 1.1%	0.79 (0.2–3.18)	0.71 (0.18–2.84)	0.625
Preterm delivery	63 28.4%	653832 7.2%	5.12 (3.82–6.85)	**4.73** **(3.53–6.36)**	0.001
Abruptio placenta	<11	97474 1.1%	2.13 (0.88–5.16)	0.77 (0.73–4.31)	0.209
Chorioamnionitis	<11	165323 1.8%	1.76 (0.83–3.73)	1.92 (0.90–4.07)	0.091
Operative vaginal delivery	<11	489398 5.4%	0.241 (0.08–0.75)	**0.27** **(0.09–0.84)**	0.024
CS	164 73.9%	2939754 32.3%	5.92 (4.39–7.99)	**5.40** **(4.00–7.30)**	0.001
SVD	55 24.8%	5667414 62.3%	0.20 (0.15–0.27)	**0.21** **(0.16–0.29)**	0.001
Hysterectomy	62 27.9%	7037 0.1%	498.02 (371.11–668.33)	**390.23** **(286.43–531.65)**	0.001
PPH	<11	263960 2.9%	0.77 (0.32–1.87)	0.768 (0.32–1.86)	0.559
Wound complications	<11	32731 0.4%	2.52 (0.63–10.13)	2.14 (0.53–8.62)	0.285
Maternal Death	0 0%	638 0.0%	NA	NA	0.996
Transfusion	39 17.6%	90328 1.0%	21.14 (14.96–29.87)	**19.23** **(13.57–27.25)**	0.001
**Other complications**					
Maternal infection	<11	199260 2.2%	1.67 (0.82–3.38)	1.80 (0.89–3.65)	0.102
DVT	<11	3831 0.0%	10.74 (1.51–76.60)	**9.42** **(1.32–67.20)**	0.025
Pulmonary embolism	<11	1658 0.0%	24.82 (3.48–177.09)	**20.22** **(2.83–144.48)**	0.003
VTE	<11	5309 10.1%	7.75 (1.09–55.26)	6.65 (0.93–47.45)	0.059
DIC	0 0.0%	18244 0.2%	NA	NA	0.996

^a^Pregnancy Outcomes: Adjusted for Age, Medical insurance Plan type, Drug use, Chronic HTN, and Tobacco Smoking in pregnancy.

^b^Delivery Outcomes: Adjusted for Age, Medical insurance Plan type, Illicit Drug use, Chronic HTN, and Smoking in pregnancy.

Note an OR and CI cannot be calculated when no cases of an occurrence occulted and as such it was presented as NA (not applicable).

Table 3 displays the association between a cervical cancer diagnosis prior to delivery and neonatal outcomes for the baby after adjusting for potential confounders, including maternal age, medical insurance plan type, illicit drug use, chronic hypertension, and tobacco smoking during pregnancy. All neonatal outcomes examined, including congenital anomalies, intrauterine fetal demise, and being small for gestational age were found to be comparable between the two groups.

**Table 3:** Comparing neonatal outcomes between cervical cancer and non-cervical cancer groups

Neonatal outcomes^a^					
Outcomes	Cervical cancer (*N*) (%)	No cervical cancer (*N*) (%)	Crude OR (95% CI)	Adjusted OR (95% CI)	Adjusted *p*-value
SGA	<11	198067 2.2%	0.62 (0.20–1.92)	0.55 (0.18–1.72)	0.305
IUFD	<11	38258 0.4%	1.07 (0.15–7.64)	0.92 (0.13–6.54)	0.930
Congenital Anomalies	0 0%	38244 0.4%	NA	NA	0.995

^a^Adjusted for Adjusted for maternal Age, Medical insurance Plan type, Illicit Drug use, Chronic HTN, and Tobacco Smoking in pregnancy.

## DISCUSSION

We have compared pregnancy, delivery, and neonatal outcomes between women with a diagnosis of cervical cancer that preceded their delivery admission and those without. Our principal observations were the following: (1) Pregnant women diagnosed with cervical cancer prior to delivery were characterized by increased maternal age, increased likelihood of having Medicare insurance, and higher rates of illicit drug use, tobacco smoking during pregnancy, and chronic hypertension; (2) Pregnant women diagnosed with cervical cancer prior to delivery had increased risks for preterm delivery, cesarean section delivery, hysterectomy, blood transfusions, deep venous thrombosis, and pulmonary embolisms, as well as decreased risks for operative vaginal deliveries and spontaneous vaginal deliveries; (3) Most pregnancy complications and delivery complications did not increase in women with cervical cancer (4) Neonatal outcomes for the babies of pregnant women diagnosed with cervical cancer prior to delivery are comparable to those of the babies of control group mothers.

**Figure 1:**
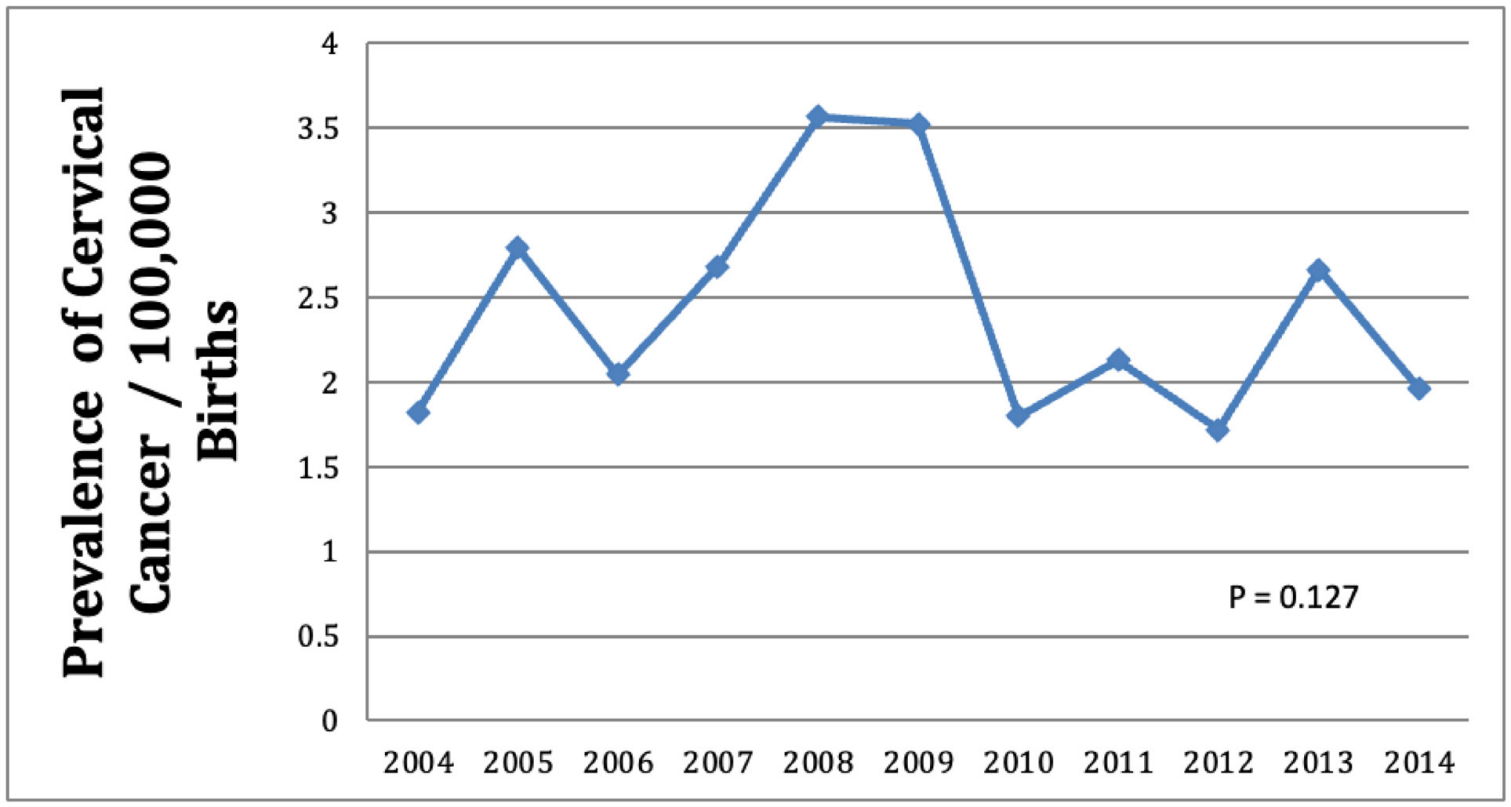
Prevalence of cervical cancer in pregnant women per 100, 000 births from 2004–2014.

The global prevalence of cervical cancer has been well-studied, with a cited age-standardized incidence of 13.3 cases per 100 000 women-years [6], the prevalence of cervical cancer diagnosis in pregnant women prior to delivery is much less well described. A previous study found the prevalence of cervical cancer diagnosis in pregnant women prior to delivery to be 0.8 cases per 10 000 pregnancies [7], higher than what we have identified (Figure 1). This discrepancy could be explained by the fact that they based their study on data gathered from 1992–1997, whilst ours was based on more recent data. It is reasonable that, over time, advances in screening/early detection, human papilloma virus vaccination and increased awareness about safe sex practices could have resulted in a decreased incidence rate. It is also possible that the rate of cervical cancer diagnosis in pregnant women prior to delivery was under-represented in our study population. However since the control group was very large, even if some women with cervical cancer were included in this group, they would fail to alter the outcomes statistically.

We found that pregnant women diagnosed with cervical cancer prior to delivery were older and more likely to have Medicare insurance. According to the National Cancer Institute, 64.9% of cervical cancer cases are diagnosed in people aged between 35 and 64 [8]. Older age is associated with cumulative cervical cancer risk factor, therefore increasing the odds of cervical cancer development with age.

We also found a higher frequency of tobacco smoking during pregnancy and illicit drug use in the cervical cancer group. This is in accordance with the known association between tobacco smoking and an increased risk for cervical cancer [9]. Through immune suppression, synergistic interaction with human papilloma virus (HPV), and hormone (estrogen and progesterone) level alteration, smoking accelerates the progression of precancerous lesions to an invasive state [9]. Similarly, illicit drug usage was proven to be correlated with risky sexual behaviors and associated HPV infection, therefore putting illicit drug users at an increased risk for cervical cancer [10].

Within our cohort, there was a significantly increased rate of chronic hypertension among women in the cervical cancer group. This aligns with the findings of several other studies. A recent study identified a positive association between hypertension and local invasion in early cervical cancer [11]. Another study found that metabolic syndromes (hypertension included) were associated with an increased risk of persistent cervical HPV infection [12], further explaining the positive relationship between hypertension and cervical cancer. It is theorized that metabolic syndromes increase the risk of HPV infection or persistence due to elevated levels of estrogen (seen in obesity or insulin resistance) and inflammatory cytokines. The inflammation inherent in the metabolic syndrome minimizes the body’s ability to fight infection likely including HPV [11, 12]. Moreover, hypertension is also associated with a faster progression of cervical cancer [11, 13]. According to a study conducted in China, patients with hypertension and cervical cancer were found to have an increased risk of parametrial invasion and poorer overall survival rates [11].

Regarding the mode of delivery, women in the cervical cancer group had increased risks for preterm delivery and decreased risks for operative vaginal deliveries and spontaneous vaginal deliveries. The increased prevalence of preterm deliveries is most likely iatrogenic with deliveries being induced to then offer more definitive cervical cancer treatments. It may also be explained by the results seen in an American study that found that patients who were screened more often for cervical cancer are at an increased risk for non-iatrogenic preterm delivery during pregnancy [13]. Another study with similar findings suggests that cervical conization or trachelectomy, a method used to both diagnose and treat cervical cancer, is associated with an increased risk of preterm delivery [14]. Sadly, the data does not permit us to know if the preterm deliveries were iatrogenic or pathologic, however, we can expect that both mechanisms contributed to these outcomes.

Regarding the hemodynamic effects of cervical cancer during pregnancy, women in the cervical cancer group were found to have an increased incidence of deep venous thrombosis, pulmonary embolisms, and blood transfusion requirements. This is in alignment with findings of a study that found that all gynecological cancers are associated with an increased risk for venous thromboembolic events, among them cervical cancer having the strongest association [15]. This can be attributed to cancer altering the body’s physiological state, engendering a hypercoagulable and inflammatory environment and thereby contributing to clot formation and thromboembolic events. Since a cervix with malignancy is more likely to hemorrhage [16], particularly in the presence of a vaginal delivery (which was the mode of delivery in a quarter of subjects in this study), it is not surprising that blood transfusions were increased. The number of cases with thromboembolic events were small and results should be confirmed in a larger study however, results were in line with the expected pathophysiology.

Importantly, except for the anticipated results in the group with cervical cancer of increased risks of cesarean section (which is often the preferred manner of delivery in these subjects), and the collateral decrease in vaginal deliveries, the risk of thromboembolic events, which is a known complication of malignancy, the increase in blood transfusions and hysterectomy to treat the underlying carcinoma, other pregnancy complications did not increase. It could have been hypothesized that the anticipated vascular alterations in carcinoma may have increased risks of hypertensive disorders of pregnancy, placental abruption, and placenta previa however, this did not occur. The results seen in the offspring was also reassuring. with no increased risks of fetal demise, small for gestational age or congenital anomalies despite that some mothers must have gotten radiation therapy during the pregnancy. Although, we are limited by the fact that the HCUP database does not permit us to detect how many of these subjects receipted this care and exactly what treatment of the cervical cancer occurred during the pregnancy.

Our study exhibits several noteworthy limitations. First off, the study is retrospective, limiting its ability to establish direct causality. Retrospective studies are observational in nature and permit us to form associations, but not direct cause-and-effect relationships. Retrospective studies are also inherently limited by the accuracy and completeness of the data that is recorded. While the HCUP-NIS database is extensive, it is based on hospital discharge data, and as such may be subject to missing information or inaccuracies in the recording of patient diagnoses, procedures, and outcomes. The database also does not include information on the treatments that the women received for their cervical cancer. These treatments can significantly impact both maternal and fetal outcomes and the lack of detailed treatment data limits the ability to assess how different therapeutic approaches influence the studied outcomes. Moreover, the study focused only on pregnancies at 24 weeks gestation or beyond, excluding early pregnancies that may have ended in miscarriage or were too early for a viable pregnancy. It is also important to consider the potential for sampling bias, as the cervical cancer group had a higher prevalence of Medicare insurance and illicit drug use, which could influence health outcomes and access to care, despite being controlled for in the multivariate logistic regression. Data on cancer stage was not available. Limited generalizability due to exclusive use of an American database. Neonatal outcomes may be underpowered due to low event rates. The HCUP does not provide the ability to investigate long-term neonatal outcomes and childhood development. Lastly, although it is one of the largest studies on this subject to date, the sample size of pregnant women with cervical cancer was small (*n* = 222) and it is possible their data does not represent the entire population.

Nonetheless, our study has numerous strengths. First off, the HCUP-NIS database provides a substantially large study sample size which augments the statistical power of our study and increases likelihood of our findings applying beyond the study population. This large dataset also enables us to adjust for numerous potential confounders, including demographic, medical, and behavioral factors, providing more reliable estimates of associations compared to smaller or less detailed studies. Furthermore, the fact that our study uses a population-based-cohort further contributes to its accurate real-world representation. It is one of the largest studies on women with cervical cancer on pregnancy complications to date. Moreover, while previous studies often focus on either maternal or neonatal outcomes, our study is one of the few to comprehensively analyze both. This dual focus enables a holistic understanding of the impact of cervical cancer during pregnancy on all facets of maternal and neonatal health.

In summary, cervical cancer during pregnancy presents significant yet expected risks for both maternal and delivery outcomes. Effective management requires a multidisciplinary team specializing in oncological, obstetrical, and neonatal care. Preconception counseling should address co-morbidities and prior medical treatments to optimize outcomes. Timely follow-up and prompt treatment during pregnancy remain critical.

Future research should focus on evaluating the impact of cervical cancer treatments during pregnancy to provide further insights into optimizing care. Additionally, given the low rates of adverse neonatal outcomes observed in this study, investigations into long-term neonatal health and childhood development are warranted to understand the broader implications of cervical cancer in pregnancy. An international study examining these factors would help determine the generalizability of these findings beyond the U.S. population.

Reassuringly, apart from the anticipated changes in pregnancy-related risks, no other significant complications were observed in women with cervical cancer during pregnancy. These findings offer a measure of reassurance while emphasizing the need for continued research and individualized care in this unique patient population.

## MATERIALS AND METHODS

This study is a retrospective analysis of a population-based cohort, utilizing the Healthcare Cost and Utilization Project Nationwide Inpatient Sample (HCUP-NIS) database [17]. It stands as the largest inpatient sample database in the United States of America and includes hospital inpatient stays submitted by healthcare facilities across the nation. On an annual basis, this database provides detailed information on seven million inpatient stays, encompassing a wide range of details such as patient characteristics, diagnoses, and procedures. The NIS is drawn from all States participating in HCUP, covering more than 97 percent of the U.S. population. The NIS approximates a 20-percent stratified sample of discharges from U.S. hospitals, excluding rehabilitation and long-term acute care hospitals, spanning across 48 states and the District of Columbia. We included in the database all women who delivered or had a maternal death between 2004 and 2014, ensuring that each pregnancy was included only once. Notably, our data included only a possibly viable pregnancy at 24 weeks or above and did not include earlier miscarriages. The database was changed to ICD-10 codes in 2015 which are not compatible with ICD-9 Codes preventing prolongation of the study duration.

The cohort was divided into two groups according to cervical cancer diagnosis – women diagnosed with cervical cancer before or during pregnancy (study group) and women without a cervical cancer diagnosis (control group). The patient’s CC diagnosis was categorized based on an International Classification of Diseases 9th Revision (ICD-9) diagnosis code 180.0, 180.1, 180.8, and 180.9. Since most women with cervical cancer are treated with hysterectomy, or significant pelvic radiation therapy making the uterus unbale to carry a pregnancy, the group studied would consist of women diagnosed in pregnancy or who were diagnosed pre-pregnancy and were treated with trachelectomy and instructed to undergo a rapid course to pregnancy. The collected data comprised a range of demographic and obstetric parameters, labor-related details, and short-term maternal and neonatal outcomes up to the point of discharge. Baseline clinical characteristics included: obesity (defined as a body mass index greater than or equal to 30 kg/m^2^); tobacco smoking during pregnancy; chronic hypertension; previous cesarean delivery (CD); pregestational diabetes mellitus (DM); thyroid disease; multiple gestations; *in-vitro* fertilization (IVF), and illicit drug use.

Pregnancy and delivery outcomes included: preeclampsia; eclampsia; pregnancy-induced hypertension; gestational hypertension; placenta previa; placental abruption; gestational diabetes mellitus (GDM); preterm premature rupture of membranes (PPROM); preterm delivery (<37 weeks); operative vaginal delivery; CD; placental abruption; chorioamnionitis; hysterectomy; postpartum hemorrhage (PPH); maternal infection; maternal death; need for blood transfusion; disseminated intravascular coagulation (DIC); deep vein thrombosis; pulmonary embolism and venous thromboembolism (VTE). Neonatal outcomes examined included: small-for-gestational-age (SGA) neonates; congenital anomalies and intra-uterine fetal death (IUFD).

Statistical analysis was performed using SPSS 23.0 (IBM Corporation, Chicago, USA). The overall prevalence of pregnant women diagnosed with CC was ascertained and then the differences in baseline characteristics between women with a diagnosis of AS and those without were compared using the chi-squared test. Subsequently, univariate and multivariate logistic regression analyses were conducted to evaluate the unadjusted and adjusted effects, respectively, of a CC diagnosis on maternal and neonatal outcomes, estimating odds ratios (OR) and 95% confidence intervals (CI). The regression models were adjusted to account for potential confounding factors, including maternal demographics, pre-existing clinical characteristics, and concurrently occurring conditions in which the chi-squared tests had shown significance (*P* < 0.05).

This study exclusively utilized publicly accessible, anonymized data. As a result, in accordance with articles 2.2 and 2.4 of the Tri-Council Policy Statement (2010), institutional review board approval was not required [18].

Data accessibility information: the HCUP database is publicly available and as such the data used in this study is publicly available.

Note per HCUP protocol when less than 11 subjects were present in any group then they should be represented as <11 to maintain patient anonymity. Unless 0 patients were affected in a group, were there would be as such no patient anonymity issues and therefore could be represented as 0 cases.

## Abbreviations

CC: cervical cancer
OR: odds ratio
CI: confidence interval
HMO: Health Maintenance Organization
CS: cesarean section
HTN: hypertension
DM: diabetes mellitus
HIV: human immunodeficiency virus
IVF: *in vitro* fertilization
GDM: gestational diabetes mellitus
PPROM: preterm premature rupture of membranes
SVD: spontaneous vaginal delivery
PPH: postpartum hemorrhage
DVT: deep vein thrombosis
VTE: venous thromboembolism
DIC: disseminated intravascular coagulation
SGA: small for gestational age
IUFD: intrauterine fetal demise

## References

[1] World Health Organization. Cervical cancer. World Health Organization. 2021. Accessed March 15, 2024. https://www.who.int/news-room/fact-sheets/detail/cervical-cancer.

[2] Zhang S, Xu H, Zhang L, Qiao Y. Cervical cancer: Epidemiology, risk factors and screening. Chin J Cancer Res. 2020; 32:720–28. 10.21147/j.issn.1000-9604.2020.06.05. 33446995 PMC7797226

[3] He Z, Xie C, Qi X, Hu Z, He Y. The effect of preserving pregnancy in cervical cancer diagnosed during pregnancy: a retrospective study. BMC Womens Health. 2022; 22:314. 10.1186/s12905-022-01885-w. 35879712 PMC9317436

[4] Ma J, Yu L, Xu F, Yi H, Wei W, Wu P, Wu S, Li H, Ye H, Wang W, Xing H, Fan L. Treatment and clinical outcomes of cervical cancer during pregnancy. Ann Transl Med. 2019; 7:241. 10.21037/atm.2019.04.76. 31317011 PMC6603350

[5] Kyrgiou M, Mitra A, Arbyn M, Stasinou SM, Martin-Hirsch P, Bennett P, Paraskevaidis E. Fertility and early pregnancy outcomes after treatment for cervical intraepithelial neoplasia: systematic review and meta-analysis. BMJ. 2014; 349:g6192. 10.1136/bmj.g6192. 25352501 PMC4212006

[6] Singh D, Vignat J, Lorenzoni V, Eslahi M, Ginsburg O, Lauby-Secretan B, Arbyn M, Basu P, Bray F, Vaccarella S. Global estimates of incidence and mortality of cervical cancer in 2020: a baseline analysis of the WHO Global Cervical Cancer Elimination Initiative. Lancet Glob Health. 2023; 11:e197–206. 10.1016/S2214-109X(22)00501-0. 36528031 PMC9848409

[7] Smith LH, Dalrymple JL, Leiserowitz GS, Danielsen B, Gilbert WM. Obstetrical deliveries associated with maternal malignancy in California, 1992 through 1997. Am J Obstet Gynecol. 2001; 184:1504–12. 10.1067/mob.2001.114867. 11408874

[8] Healthline. What Age Does Cervical Cancer Typically Occur? Healthline. Accessed March 15, 2024. https://www.healthline.com/health/cervical-cancer/what-age-cervical-cancer#age-and-diagnosis

[9] National Cancer Institute. Cervical Cancer Prevention (PDQ®)–Patient Version. National Cancer Institute. 2022. Accessed March 15, 2024. https://www.cancer.gov/types/cervical/hp/cervical-prevention-pdq

[10] Sharma S, Al Daghreer RAA, Aldaghreer H. Cancer Association in Drug Abuse Disorders: A latest Update. Adv Can Res & Clinical Imag. 2022; 3. 10.33552/ACRCI.2022.03.000569.

[11] Shen T, Zhao J, Li W, Wang X, Gao Y, Wang Z, Hu S, Cai J. Hypertension and hyperglycaemia are positively correlated with local invasion of early cervical cancer. Front Endocrinol (Lausanne). 2023; 14:1280060. 10.3389/fendo.2023.1280060. 38152132 PMC10752498

[12] Huang X, Zhao Q, Yang P, Li Y, Yuan H, Wu L, Chen Z. Metabolic Syndrome and Risk of Cervical Human Papillomavirus Incident and Persistent Infection. Medicine (Baltimore). 2016; 95:e2905. 10.1097/MD.0000000000002905. 26945384 PMC4782868

[13] Bromley-Dulfano RA, Rossin-Slater M, Bundorf MK. Association Between Cervical Cancer Screening Guidelines and Preterm Delivery Among Females Aged 18 to 24 Years. JAMA Health Forum. 2023; 4:e231974. 10.1001/jamahealthforum.2023.1974. 37477927 PMC10362467

[14] Kasuga Y, Ikenoue S, Nishio H, Yamagami W, Ochiai D, Tanabe K, Tashima Y, Hirao N, Miyakoshi K, Kasai K, Suda Y, Nemoto T, Shiraishi S, et al. Adenocarcinoma *in situ* or early-stage cervical cancer is a risk factor for preterm delivery after cervical conization: a multicenter observational study. J Matern Fetal Neonatal Med. 2022; 35:9837–42. 10.1080/14767058.2022.2056835. 35341455

[15] Falanga A, Lorusso D, Colombo N, Cormio G, Cosmi B, Scandurra G, Zanagnolo V, Marietta M. Gynecological Cancer and Venous Thromboembolism: A Narrative Review to Increase Awareness and Improve Risk Assessment and Prevention. Cancers (Basel). 2024; 16:1769. 10.3390/cancers16091769. 38730721 PMC11083004

[16] Eleje GU, Eke AC, Igberase GO, Igwegbe AO, Eleje LI. Palliative interventions for controlling vaginal bleeding in advanced cervical cancer. Cochrane Database Syst Rev. 2015; 2015:CD011000. 10.1002/14651858.CD011000.pub2. 25932968 PMC6457846

[17] Steiner C, Elixhauser A, Schnaier J. The healthcare cost and utilization project: an overview. Eff Clin Pract. 2002; 5:143–51. 12088294

[18] Canadian Institutes of Health Research, Natural Sciences and Engineering Research Council of Canada, and Social Sciences and Humanities Research. Council, Tri-Council Policy Statement: Ethical Conduct for Research Involving Humans. 2018.

